# First Commencement of the Med. Department of the University of Buffalo

**Published:** 1847-07

**Authors:** 


*First Commencement of the Med. Department of the University of Buffalo.*—The first commencement of the above Institution was held on the 16th ult., in the First Presbyterian Church, of this city.

The Exercises commenced with a fervent prayer, by the Rev. Mr. Hopkins, Pastor of the First Presbyterian Church. This was followed by Music, by the Choir of the Church. Hon. Mr. Fillmore, Chancellor of the University, then delivered an address, giving an historical account of the educational enterprises heretofore undertaken here—the rise and progress of this institution, and urging, in an emphatic, forcible manner its claims upon the liberality of our citizens,in order that its character and permanency may be secured.

Next followed Music by the Choir; when the Chancellor conferred the degree of M. D. upon the following gentlemen :—

Henry D. Garvin, Buffalo, N. Y.
John P. Dudley, Buffalo, N. Y.
George Bruce Parker, Trenton, N. Y.
John M. Hardy, Canada West.
James Albert Whiting, Canada West.
Samuel Culver Rogers, Cortland, N. Y.
William Ring, Tompkins, N. Y.
Miron Holmes Andrews, Eaton, Mich.
Zara Hurd Blake, Dansville, N. Y.
Wells Taber, Honeoye, N. Y.
Bela E. Phelps, Herkimer, N. Y.
Sidney Aldridge Foss, Mount Washington, Ky.
Horace Stewart Lindley, Mansfield, Ohio.
J. E. King, Warren, Pa.
Henry W. Bennett, Jamestown, N. Y.
Delois M. Norton, Covington, N. Y.
George Abbott, New York, N. Y.

After the diplomas were conferred, Professor Hamilton delivered an impressive and eloquent charge to the graduates.

The Exercises concluded by Music by the Choir, and benediction by the Rev. Mr. Schuyler, of St. John's Church, of this city.

We have thus chronicled not only the first commencement of the Medical College, but the first event of the kind which has ever transpired in our flourishing city. The occasion was one of much public enthusiasm, and was justly characterised by the Chancellor, in his address, as marking an era in the history of Buffalo. The general interest manifested, from the first, in our youthful institution, has been greatly enhanced by the encouraging success which has thus far crowned the enterprise. So far as we know, no Medical Institution in our country, with a single exception, ever commenced with so large a class as attended the first session of this school. Efforts will be at once made to secure, by the time the lease of the present building expires, the erection of a commodious edifice ; and we feel that we are justified in anticipating for the institution, the speedy and full realization of the most sanguine expectations both of the public and profession.

Agreeably to the request of the graduates, the addresses by Mr Fillmore and Professor Hamilton have been published in the *Commercial Advertiser*, of this city.

